# Associating serum testosterone levels with African ancestral prostate cancer health disparities

**DOI:** 10.1038/s41598-025-92539-y

**Published:** 2025-04-08

**Authors:** Maphuti Tebogo Lebelo, Naledi Mmekwa, Melanie Louw, Weerachai Jaratlerdsiri, Shingai B. A. Mutambirwa, Massimo Loda, Vanessa M. Hayes, M. S. Riana Bornman

**Affiliations:** 1 Department of Biochemistry, Genetics and Microbiology, University of Pretoria, Pretoria, South Africa; 2 Department of Physiology, Faculty of Health Sciences, University of Pretoria, Pretoria, South Africa; 3 School of Health Systems & Public Health, University of Pretoria, Pretoria, South Africa; 4 National Health Laboratory Services, Johannesburg, South Africa; 5 Department of Anatomical Pathology, School of Pathology, University of the Witwatersrand, Johannesburg, South Africa; 6 Ancestry and Health Genomics Laboratory, Faculty of Medicine and Health, School of Medical Sciences, Charles Perkins Centre, University of Sydney, Camperdown, NSW Australia; 7 Department of Urology, Dr George Mukhari Academic Hospital, Sefako Makgatho Health Science University, Medunsa, South Africa; 8 Department of Pathology and Laboratory Medicine, Weil Cornell Medicine, New York Presbyterian-Weill Cornell Campus, New York, NY USA; 9 Manchester Cancer Research Centre, University of Manchester, Manchester, M20 4GJ UK

**Keywords:** Testosterone, African ancestry, Prostate cancer, Cholesterol, Health disparity, Prostate cancer, Prostate

## Abstract

Serum testosterone levels decrease in the aging male, while the risk for prostate cancer (PCa) increases concomitantly. Higher levels in younger men have been linked with racially driven PCa disparities, with African men disproportionately impacted. In turn, higher levels of serum lipids have been associated with aggressive disease, while racial disparity between serum testosterone, cholesterol and cancer mortality has been suggested. Having previously reported a 2.1-fold increased age-adjusted risk for aggressive PCa in Black South African over Black American men, we determined the serum testosterone and associated lipid levels in 250 Black South African men either with or without clinicopathologically diagnosed disease. Observing no associations with serum lipid levels, Black South Africans presented with testosterone levels between 1.24 (< 60 years) and 1.3-fold (≥ 60 years) greater than Black Americans. Notably, a rapid drop in total-, bioavailable- and free testosterone levels in men 65 years or older was significantly associated with PCa risk (*P* = 0.0057, 0.009 and 0.005, respectively), while irrespective of age, further associated with advanced disease (*P* = 0.004, 0.0012 and 0.0036, respectively). These preliminary data provide insights into the potential role of androgens in driving PCa health disparities, with important consequence for tailoring treatment for Black men.

**Supplementary Information:**

The online version contains supplementary material available at 10.1038/s41598-025-92539-y.

## Introduction

Prostate cancer (PCa) is characterized by significant ancestral disparity. In the United States, African American men are diagnosed at a younger age, present with more advanced disease, and have the greatest lifetime risk of dying from PCa^1,2^. With Black men at double the risk for lethal disease compared to White American men, this disparity increases to 4.2-fold for men younger than 50 years^3^. Globally, the regions of sub-Saharan Africa and the Caribbean are the most impacted by PCa mortality, with the highest rates reported for Southern Africa^4^, which is notable when considering the average life expectancy for southern African men is 1.2-fold lower than the worldwide average at birth (World Bank 2020). Previously we showed that, compared to African American men and after adjusting for age, Black South African men are 2.1-fold more likely to present with aggressive disease^5^. What is clear, African ancestry is a well-established risk factor for PCa adversity. While the reasons for this disparity are likely multifactorial, including socioeconomic and healthcare access as contributing factors, an extensive review of the literature concurred that PCa presentation in African American men is both genomically and biologically unique^6^. While data is comparably scare for Sub-Saharan Africa, most recently we demonstrated that even within the broad ‘African’ racial identifier men of southern African ancestry present with regionally unique inherited and somatic mutational profiles^7–9^. However, how these genomic differences translate into biological differences is largely unknown.

Testosterone is required for normal prostate growth, which makes androgen deprivation therapy (ADT) an obvious first-line treatment for advanced or metastatic disease^10^. However, a lack of consensus exists regarding the role of serum testosterone levels in PCa development and progression^11^. While some studies refute an association between testosterone and PCa predisposition^12–14^, others have linked high testosterone levels to elevated risk^15–17^ or irrespective of PCa pathology, elevated prostate specific antigen (PSA) levels^18^. Overall, circulating testosterone levels drop both as men age (rate of 1.6% per year) and as cancer progresses, with low levels (≤ 300 ng/dL) associated with aggressive disease^19^. For ADT treated patients, achieving chemical castration requires total serum testosterone to drop below 50 ng/dl, while levels of 25 ng/dl have been shown to have prognostic potential and patient stratification for combination therapies^20^, although castration-resistance is inevitable^21^. Notably, age-related racial variations in testosterone levels show that compared to White Americans, Black American men present with overall higher levels especially in the younger < 45-year age group^22^, although reportedly not true for adolescent males^23^, with others showing earlier and higher peaking, with more dramatic age-related decline^24^. For the PCa male, serum testosterone levels have been reported to be similar after adjusting for age and grade between Black and White American men^25,26^, emphasizing the need for further efforts to elucidate racial disparities.

As testosterone is a cholesterol-derived steroid hormone, it has been postulated that a link exists between testosterone and cholesterol levels and in turn PCa risk. Notably, hypercholesterolemia has been associated with poor PCa prognosis or risk for aggressive disease, with risk reduction concurring with the use of cholesterol-lowering statins^27–29^. While a single study reported significance between elevated cholesterol and PCa recurrence in Black over White men, triglyceride levels were found to be associated with recurrence in both races^30^. Introducing further complexity concerning serum testosterone levels and ancestry associated PCa health disparities, decreased testosterone levels has been shown to elevate the risk of cardiovascular disease in Black men, with an inverse association observed for White Americans^31^. Most recently, irrespective of race and after adjusting for potential cofounding factors, serum cholesterol and testosterone levels were not associated in a study of 1996 American men^32^.

Despite decades of research, there is a dearth of data for the African continent. In this study, we assessed serum testosterone, cholesterol and triglyceride levels against PSA levels and clinicopathological presentation in a cohort of 250 self-identified Black South African men, distinguishing between those with treatment naïve localized disease, and those without PCa. Compared with published data for Black Americans, we found testosterone levels to be higher in Black South African men, with age-related decline pronounced for men with PCa. Acknowledging our small study size, the power lies in highlighting the importance of expanding efforts across Sub-Saharan Africa if we are to understand the clinical significance of biological contributions to PCa health disparities within the continent.

## Results

### Clinicopathological presentation, PSA validation and ancestry

All recruited men visited a Southern African Prostate Cancer Study (SAPCS) urology clinic between 2018 and 2019 due to urological complaints, including erectile dysfunction, lower urinary tract symptoms, swollen scrotal region, dysuria or hematuria, and were recommended for prostate biopsy. The mean age at presentation was 66.5 years for patients with PCa and 66.2 years for controls. Through histopathological analyses of 12 or more biopsy cores per patient, PCa diagnosis was confirmed via Gleason score and International Society of Urologic Pathologists (ISUP) group grading, resulting in 120 men receiving a positive diagnosis (case) and 130 a negative diagnosis (control; Table 1). Patients with PCa were further grouped according to their ISUP grading (or Gleason score) as high-risk (HRPCa, ISUP 3–5, *n* = 51) or low-risk (LRPCa, ISUP 1–2, *n* = 69) PCa. For men without PCa, 18.5% (*n* = 24) presented with benign prostatic hyperplasia (BPH), 30.8% (*n* = 40) with prostatitis and 17.7% (*n* = 23) presented with fibromuscular hyperplasia. Conversely, prostatitis co-occurred in 4.2% (*n* = 5) of PCa cases, with no sign of BPH or fibromuscular hyperplasia.

**Fig. 1:**
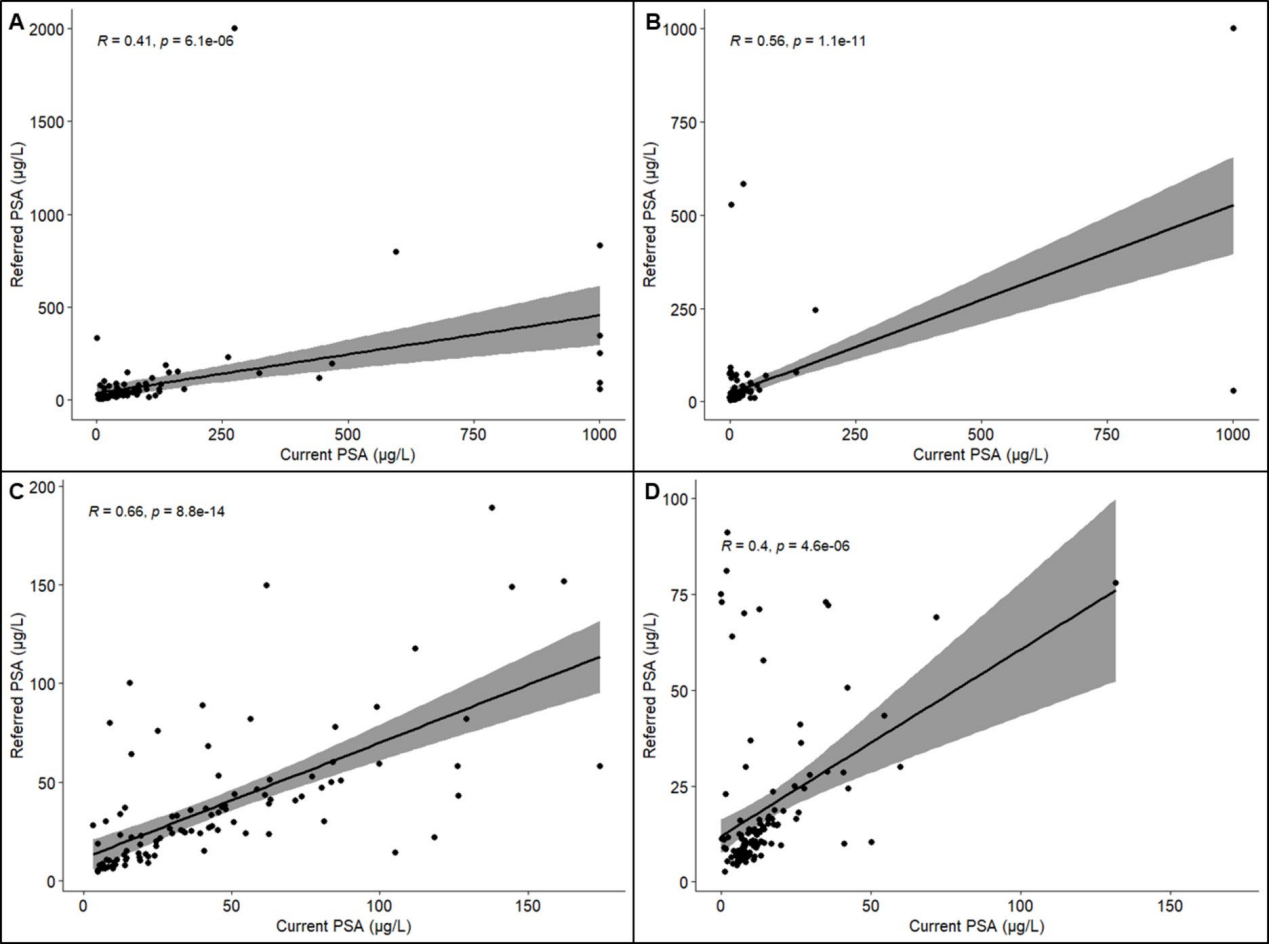
Scatterplot of PSA levels at referral (y-axis) and the second single laboratory or current PSA (x-axis) of Black South African men with (*n* = 120) and without (*n* = 130) prostate cancer (PCa). PSA scatter plot of men with (**A**) and without (**B**) PCa. PSA levels < 200 µg/L for men with (**C**) and without (**D**) PCa.

As expected, PSA levels were significantly elevated in cases *versus* controls (102.4 vs. 31.4 µg/L, *P* < 0.001) (Table 1) and in HRPCa *versus* LRPCa and no PCa groups (Fig. S1A). Concurring with previous data reported for Black South African men^5^, PSA levels far exceeded global averages for both groups, while the difference appears greatest for men with PSAs ≥ 20 µg/L (68.4% cases vs. 21.6% controls, *P* = 0.009; Fig. S1B). Significance remained true after exclusion for extreme PSA outliers (Fig. S1D, E), identified using the interquartile range method. Notably, the largest racial-based study out of the United States showed Black men without PCa to have the highest PSA levels compared to all other racial groups^33^. Here, we provide experimental validation for elevated PSA levels through a second PSA screen performed in a single laboratory (current PSA) for both cases and controls (Table 1), with a correlation coefficient of 0.41 (Fig. 1A) and 0.56 (Fig. 1B), respectively, and 0.66 (Fig. 1C) and 0.4 (Fig. 1D) when excluding for PSA > 200 µg/L. While showing statistically significant correlations, we acknowledge that the discordant calls in the control group are more likely to be driven by an exaggerated referral PSA. Appreciating that controls with maintained current PSA levels ≥ 20 µg/L (20.8%) may represent either a misdiagnosis or an associated hyperplasia or infection, we tested for the latter. However, we found no difference in the presence of prostatitis between controls with PSA levels < 20 (29.4%, 30/102) and ≥ 20 µg/L (28.6%, 8/28), while BPH was more prominent in men with PSA < 20 (20.6%, 21/102) than ≥ 20 µg/L (10.7%, 3/28).

We further defined African ancestry through two generational self-identification, with the inclusion of one or more of the following southern Bantu or Black South African ethno-linguistic groupings defined via Guthrie Zone-S linguistic groups as S20-Venda (Tshivenda speakers), S30-Sotho-Tswana (Sesotho, Sepedi, Setswana), S40-Nguni (isiNdebele, isiXhosa, isiZulu, siSwazi), and S50-Tsonga (Xitsonga, including in our study ethnically reported Shangaan). Observing no significant clinicopathological, including PSA levels overall (Fig. S1C) and with the exclusion of extreme outliers (Fig. S1F), between the ethno-linguistic groups, with Sotho-Tswana speakers representing the largest study contributors (52.4%, 131/250; Table 1) participants were classified in downstream analyses as Southern Bantu or Black South African.

**Table 1:** Clinical and hormone/lipid biochemical characteristics of the study cohort of 250 black South African men, either with (positive) or without (negative) clinicopathologically confirmed prostate cancer.

	PCa positive (*n* = 120)	PCa negative (*n* = 130)	*P*-value
Age			
Number	119	128	0.79
Mean years (range)	66.5 (43–89)	66.2 (45–91)	
Ethno-linguistic identifier			
Venda	16 (13.3%)	10 (7.7%)	
Sotho-Tswana	69 (57.5%)	62 (47.7%)	
Nguni	20 (16.7%)	29 (22.3%)	
Tsonga	9 (7.5%)	22 (16.9%)	
Other	3 (2.5%)	5 (3.8%)	
Unknown	3 (2.5%)	2 (1.5%)	
PSA current			
Mean µg/L ± SD	102.4 ± 208.2	31.4 ± 123.6	< 0.0001
Number < 4 µg/L	2 (1.67%)	18 (13.9%)	
Number 4–9.9 µg/L	16 (13.3%)	48 (36.9%)	
Number 10–19.9 µg/L	20 (16.7%)	36 (27.7%)	
Number 20–99 µg/L	59 (49.2%)	24 (18.5%)	
Number ≥ 100 µg/L	23 (19.2%)	4 (3.1%)	
PSA referral			
Mean µg/L ± SD	81.5 ± 218.8	37.3 ± 113.5	< 0.05
Number < 4 µg/L	0 (0%)	1 (0.8%)	
Number 4–9.9 µg/L	16 (13.3%)	48 (36.9%)	
Number 10–19.9 µg/L	19 (15.8%)	44 (33.8%)	
Number 20–99 µg/L	61 (50.9%)	28 (21.5%)	
Number ≥ 100 µg/L	16 (13.3%)	4 (3.1%)	
Unknown	8 (6.7%)	5 (3.8%)	
Pathological features			
LRPCa (ISUP 1 & 2)	69 (57.5%)	–	
HRPCa (ISUP 3, 4 & 5)	51 (42.5%)	–	
BPH	–	24 (18.5%)	
Prostatitis	5 (4.2%)	40 (30.8%)	
Fibromuscular hyperplasia	–	23 (17.7%)	
Atypical acinar proliferation	–	11 (8.5%)	
Total testosterone			
Mean ng/dL ± SD	525.6 ± 308.4	574.6 ± 286.5	0.19
Number < 300 ng/dL	24 (20%)	21 (16.2%)	
Number 300–1000 ng/dL	91 (75.8%)	100 (76.9%)	
Number > 1000 ng/dL	5 (4.2%)	9 (6.9%)	
Cholesterol			
Mean mmol/L ± SD	5.1 ± 1.2	5.2 ± 1.3	0.58
Number < 5.17 mmol/L	68 (56.7%)	72 (55.4%)	
Number 5.17–6.18 mmol/L	34 (28.3)	39 (30%)	
Number > 6.18 mmol/L	18 (15%)	19 (14.6%)	
Triglycerides			
Mean mmol/L ± SD	1.66 ± 0.81	1.66 ± 0.81	0.95
Number < 1.7 mmol/L	75 (62.5%)	82 (63.1%)	
Number > 1.7 mmol/L	45 (37.5%)	48 (36.9%)	

*BPH* benign prostatic hyperplasia, *PCa* prostate cancer, *LRPCa* low-risk PCa, *HRPCa* high-risk PCa, *PSA* prostate specific antigen, *SD* standard deviation.

### Testosterone and lipid levels, against age PCa status and PSA levels

To control, as best as possible, for naturally occurring daily fluctuations in hormonal levels, all men were sampled prior to 10h00. Testing included total serum testosterone, albumin and sex hormone binding globulin (SHBG) to determine free and bioavailable testosterone, as well as serum cholesterol and triglyceride levels. Overall, 18% of the study population had low (< 300 ng/dL) total testosterone, and 5.6% had high (> 1000 ng/dL) total testosterone (Table 1). Men with PCa (525.6 ± 308.4 ng/dL) did not have significantly lower testosterone levels than men without PCa (574.6 ± 286.5 ng/dL). The mean cholesterol and triglyceride levels were similar or no different between men with and without PCa (5.1 ± 1.2 vs. 5.2 ± 1.3 mmol/L; and both 1.66 ± 0.81 mmol/L, respectively). The proportion of patients with high cholesterol levels (> 6.18 mmol/L) did not differ significantly between PCa patients and healthy controls (15% vs. 14.6%).

As PSA production is hormone dependent^34^, it is not surprising that both testosterone and cholesterol have been associated with PSA levels^18,35^. However, the latter study reported a correlation with White and not Black men prior to use of cholesterol lowering statins. Using current PSA levels, we show no correlation with testosterone in Black South African men with (*R* = − 0.066, *P* = 0.48, Fig. S2A) and without PCa (*R* = − 0.0097, *P* = 0.91, Fig. S2B), nor with cholesterol for men with (*R* = − 0.13, *P* = 0.15, Fig. S2C) and without PCa (*R* = − 0.089, *P* = 0.42, Fig. S2D). Although not significant, overall PSA and associated cholesterol levels were greater for PCa cases over controls (Fig. S3A and PSA log transformed Fig. S3B).

**Fig. 2:**
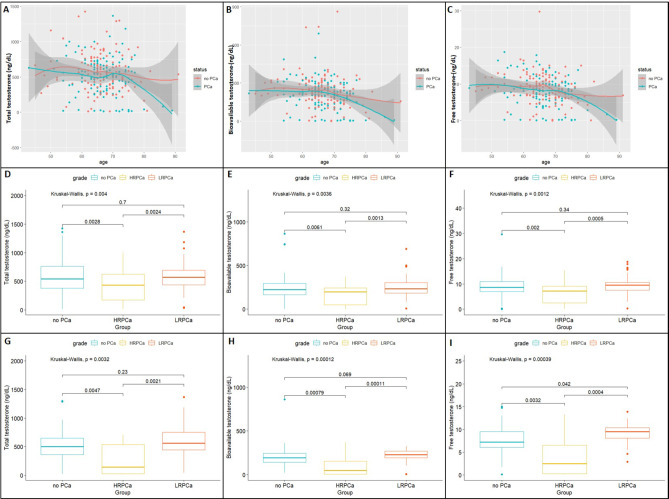
LOESS curves and boxplots of serum testosterone levels according to age and prostate cancer (PCa) status in black South African men with (*n* = 120) or without (*n* = 130) prostate cancer, including high-risk (HRPCa) or low-risk (LRPCa) disease defined by pathology. LOESS curve by age for total testosterone **(A)**, bioavailable testosterone **(B)** and free testosterone **(C)**. Boxplot of PCa status and risk for total testosterone **(D)**, bioavailable testosterone **(E)**, and free testosterone **(F)**, and restricted for men > 70 years for total testosterone **(G)**, bioavailable testosterone **(H)**, and free testosterone **(I)**,

While total (Fig. 2A), bioavailable (Fig. 2B), and free (Fig. 2C) testosterone decreased with age as expected, this decrease appears to become more pronounced for cases over controls after 70 years of age. To optimize between group correlations, we set the younger age threshold to < 65 years, one year less than the study mean. Compared with younger men, men aged $\:\ge\:$65 years with PCa had significantly lower levels of bioavailable (340.42 vs. 188.40 ng/dL, *P* = 0.045) and free testosterone (12.54 vs. 7.37 ng/dL ng/dL, *P* = 0.048), while total testosterone levels did not quite reach significance (Table 2). Moreover, compared to men without PCa, younger Black South Africans with PCa presented with a 1.33-fold and 1.39-fold higher free and bioavailable testosterone, respectively (Table S1). This is in stark contrast to older men, where free and bioavailable testosterone levels were 1.29-fold and 1.31-fold higher than controls over cases, respectively. Irrespective of age, patients presenting with high-risk (ISUP 3–5) PCa had significantly lower total (Fig. 2D), bioavailable (Fig. 2E), and free testosterone levels (Fig. 2F) than the low-risk (ISUP 1–2) patients and the control group (*P* = 0.004, *P* = 0.0036, and *P* = 0.0012, respectively). The latter holding true for men $\:\ge\:$65 years (*P* = 0.0057 (Fig. S4A), *P* = 0.009 (Fig. S4B), and *P* = 0.005 (Fig. S4C), respectively), which further increases in significance increasing for men $\:\ge\:$70 years of age (*P* = 0.0032 (Fig. 2G), *P* = 0.00012 (Fig. 2H), and *P* = 0.00039 (Fig. 2I), respectively). Of note, men presenting with LRPCa at $\:\ge\:$70 years of age have higher bioavailable testosterone compared to HRPCa men (*P* = 0.00011) and men without PCa (*P* = 0.069), with free testosterone significantly higher for LRPCa men compared to HRPCa men (*P* = 0.0004) and men without PCa (*P* = 0.042). Unlike testosterone, neither cholesterol nor triglyceride levels were linked to age-associated PCa status (Fig. S5A, B, respectively) or disease presentation (Fig. S5C, D, respectively). Additionally, no correlation was observed between total testosterone and cholesterol levels according to PCa status (Fig. S6A) or age (Fig. S6B).

### Age-associated testosterone levels between black South African and American men

According to the US-based National Health and Nutritional Examination Survey (NHANES), healthy Black men (*n* = 355) have on average 1.2-fold higher serum testosterone levels than White American men (*n* = 631), with testosterone levels appearing to decrease more rapidly for African American men^24^. As such, NHANES provides a foundation for comparative analyses for our Black South African control data (*n* = 130). Appreciating that the NHANES study uses an upper younger age associated threshold of 60 years, while we observe largely an incremental difference in testosterone levels between White and Black American and Black South African men (Table 3). Notably, this increase is greatest for men 60 years of age and over, with testosterone levels in Black South African men significantly higher than that of Black (*P* = 4.128e-12) and White (*P* = 5.82e-13) American men. While the fold difference from 40 to 59 years to $\:\ge\:$ 60 years is comparable for total (1.29) and free testosterone (1.28) for Black South African *versus* Black American men, the greatest difference was observed for bioavailable testosterone for Black South African men at 1.54-fold (Table 3) and 1.80-fold (data not shown) compared with Black and White American men, respectively. Strikingly and in contrast to both White and Black Americans who demonstrate a rapid age-related decline in free (1.60 and 1.49) and bioavailable testosterone (1.6 and 1.54), respectively, Black South African men show shallow decline (1.15 free and 1.14 bioavailable).

**Table 2:** Mean testosterone, cholesterol and triglyceride levels in younger (< 65 years) and older (≥ 65 years) black South African men (*n* = 250) defined by PCa status.

Age	PCa control			PCa case		
	40–64 years (*n* = 46)	≥ 65 years (*n* = 82)	*P*-value	40–64 years (*n* = 45)	≥ 65 years (*n* = 75)	*P*-value
Total T (ng/dL, 95% CI)	590.45 (496.77–690.50)	561.89 (497.16–610.57)	0.578	585.70 (486.97–690.95)	479.55 (422.23–552.78)	0.065
Free T (ng/dL, 95% CI)	9.41 (7.95–10.25)	9.50 (7.48–11.90)	0.944	12.54 (7.26–19.16)	7.37 (6.66–8.47)	0.048
Bioavailable T (ng/dL, 95% CI)	245.28 (203.08–276.65)	247.26 (196.17–306.06)	0.955	340.42 (181.21–514.25)	188.40 (171.12–225.30)	0.045
Cholesterol (mmol/L, 95% CI)	5.04 (4.66–5.26)	5.24 (4.97–5.57)	0.356	5.10 (4.73–5.43)	5.04 (4.79–5.32)	0.759
Triglycerides (mmol/L, 95% CI)	1.73 (1.52–2.07)	1.61 (1.43–1.76)	0.405	1.76 (1.52–1.93)	1.58 (1.42–1.82)	0.206

*PCa* prostate cancer, *T* testosterone, *CI* confidence interval.

**Table 3:** Mean testosterone levels (total, free and bioavailable) between black South African (SA, this study, *n* = 128 of 130) and black (*n* = 98 of 355) and white (*n* = 345 of 631) American (US, NHANES study) men without prostate cancer.

Testosterone	Ethnicity	Age 40–59 years			Age ≥ 60 years			Fold^3^
		n	ng/dL (95% CI)	Fold	n	ng/dL (95% CI)	Fold	
Total	White US	163	439 (402–478)	–	182	359 (326–395)	–	1.22
	Black US	56	524 (465–592)	1.19^1^	42	432 (374–499)	1.20^1^	1.21
	Black SA	16	649 (451–846)	1.24^2^	112	558 (507–608)	1.29^2^	1.16
Free	White US	163	8.80 (7.95–9.73)	–	182	5.50 (4.99–6.07)	–	1.60
	Black US	56	10.16 (8.97–11.52)	1.16^1^	42	6.84 (6.05–7.72)	1.24^1^	1.49
	Black SA	16	10.08 (8.28–11.88)	0.99^2^	112	8.74 (7.99–9.49)	1.28^2^	1.15
Bioavailable	White US	163	209 (189–231)	–	182	128 (116–141)	–	1.63
	Black US	56	231 (204–263)	1.11^1^	42	149 (131–170)	1.17^1^	1.55
	Black SA	16	263 (213–314)	1.14^2^	112	230 (206–254)	1.54^2^	1.14

*CI* confidence interval.

^1^Fold increased between Black and White Americans (US).

^2^Fold increase between Black South Africans (SA) and Black Americans (US).

^3^Fold decrease between younger men (40–59 years) and older men (≥ 60 years).

## Discussion

It is well established that total testosterone levels decrease progressively in the aging male^36^ and that an increase in SHBG leads to a greater decrease in free testosterone^37^. Furthermore, while the rate of decline is independent of ethnicity, overall, American men of African over European over Asian ancestry have the highest to lowest lifetime testosterone levels^24,38^. The latter mirroring ethnically-driven PCa incidence and mortality rate disparities^39,40^. Having previously reported a 2.1-fold increased age-adjusted risk for aggressive PCa at presentation for Black South African *versus* Black American men^5^, it is notable that through the inclusion of clinicopathologically confirmed non PCa Black South African men in this study we observe the highest globally reported population-based total testosterone levels for men 40 years and over (12.7 to 1424.3 ng/dL). Intriguingly, while only free testosterone levels in the younger age group (40–59 years) mirrored levels reported for Black Americans, irrespective of age, the total, free and bioavailable testosterone levels were all elevated in Black South Africans. Appreciating a time lapse of almost 20 years between our study and collection of the NHANES data, overall, the age-associated decline is lowest for Black South African men, with further significance observed for bioavailable testosterone.

In contrast, our study demonstrated that the observed decline in testosterone appears to be more pronounced in Black South African men with PCa, with fold-change more closely reflecting or slightly surpassing those reported for Black and White Americans without PCa. Specifically, for Black South African men over 65 years of age the rate of testosterone decline ranged from 1.05 to 1.22-fold for total, 0.99 to 1.70-fold for free, with the steepest decline noted for bioavailable testosterone 0.99 to 1.81-fold, for men with PCa *versus* our population matched controls. Most notably, we observed an age-dependent contrast in the direction of association between testosterone (free and bioavailable) levels and PCa status, with higher levels in younger men associated with PCa presentation, while in contrast higher levels were associated with a lack of PCa in older men. Irrespective of age at diagnosis, we found lower total, free and bioavailable testosterone levels to be significantly associated with high-risk disease presentation, which increased significantly for free and bioavailable testosterone in men ≥ 70 years. No correlations were observed with PSA levels (log transformed, data not shown).

Besides hormones, evidence is growing that lipid metabolism not only plays a critical role in PCa risk, outcomes and PSA, but also testosterone levels in the aging male. Appreciating that studies are limited, the community is divided on the direction of association between total cholesterol levels and PCa risk and/or high-risk disease^41–43^. Here we found no association between total serum cholesterol levels and either PCa risk, aggressive disease presentation or PSA levels, although overall Black South African men with PCa were more likely to present with higher correlated cholesterol and PSA levels. Notably, in a study of American men without PCa, total cholesterol and LDL levels were positively correlated with PSA levels in White but not in Black men, suggesting that the effect of cholesterol on PCa biology may differ by race^35^. As with cholesterol, the jury is also out regarding the direction of correlation with triglycerides including both positive^44^ and negative correlations with PCa risk and/or high-risk disease^45^. In turn, the NHANES study has negatively correlated serum triglycerides with PSA levels among American males^46^. Again, we found no correlation between triglycerides and PCa risk or presentation, PSA (log transformed, data not shown) or testosterone levels.

Limitations of our study include lack of fasted sampling and information regarding potential cofounding factors, most notably patient body mass index and lifetime cumulative data, as well as the significant range and elevation of PSA levels observed within our Black South African cohort. While neither the presence of hyperplasia and/or prostatitis appeared to be driving elevated PSA levels within our control population, the biological mechanisms behind significantly elevated PSA levels across the region and as previously reported^5^, requires further elucidation. Although there was no clinicopathological evidence for PCa in our control group, we cannot exclude for a possible missed multi-core biopsy sampling. Arguably underpowered, we are acutely aware that data for sub-Saharan Africa is scarce or lacking. Scanning the literature, we found a single Nigerian study (*n* = 55) which reported lower population-based testosterone levels compared to African American men, with lower levels linked to PCa status^47^, and two South African studies, the first (*n* = 109) associated low free testosterone with aggressive PCa and higher PSA levels^48^, and the second (*n* = 878) found no correlation between cholesterol and PCa status, although suggested an association between high-density lipoprotein and PCa diagnosis^49^. Furthermore, one needs to consider limitations associated with comparing studies across the African diaspora. We have previously shown SAPCS men to be genetically distinct from west, east and central African populations and in turn African Americans^9^, while alluding to within regional ancestral substructure^50^. Accordingly, while the patterns of testosterone and lipid expression across diverse populations in Sub-Saharan Africa remain to be determined, these studies will need to consider both biological and genetic diversity across the broad African identifier.

Largely aligned with a 2010 meta-analysis^51^, we found younger Black South African PCa cases to have a 1.4-fold overall higher testosterone levels compared with age- and population-matched non-cancer controls. While overall, Black South African men show a shallow age-related decline in testosterone, specifically free and bioavailable, compared with White and Black Americans, notably, we observed a significant age-associated decline in testosterone levels for Black South African PCa patients, suggesting a link with advanced disease in this population. Our study raises critical questions with respect to adopting European-biased criteria for PCa clinical management, while providing cautionary evidence for the potential limitations of a ‘universal’ (one-size-fits-all) African model. For example, the impact of significant differences in age-related hormone levels observed has yet to be investigated with respect to ADT use across the African diaspora. Taken together, our study underscores the urgency for African inclusion in addressing what may be perceived as an ‘old story’.

## Methods

### Ethics and participant recruitment

The study was approved by the University of Pretoria Faculty of Health Sciences Research Ethics Committee in South Africa (with US Federal wide assurance FWA00002567 and IRB00002235 IORG0001762), which included both project specific (HREC#58/2021) and SAPCS Consortia approval (HREC#43/2010), with research performed in accordance with the Declaration of Helsinki. Additional IRB review and approval was granted by the Human Research Protection Office of the US Army Medical Research and Development Command as part of the HEROIC PCaPH Africa1K Consortium (E03333.3a). Providing informed consent, 250 treatment-naïve Black South African men were recruited from participating SAPCS urology clinics, specifically Tshilidzini Hospital in Limpopo Province and Dr. George Mukhari Academic Hospital in Gauteng Province of South Africa. Furthermore, patients were not fasted prior to sampling.

### Clinicopathology and sampling

Blood (serum) was collected prior to 10h00. The samples were stored at − 20 °C on site and during transportation. At the University of Pretoria Laboratory, Department of Urology, the samples were thawed overnight at 2 °C and subsequently left to stand for 1 h to separate the serum. The serum samples were aliquoted into Eppendorf tubes at a volume of 400 µl and stored at − 80 °C for no more than 2 years. Total testosterone, total albumin, sex hormone binding globulin (SHBG), total cholesterol and triglyceride levels were tested at PathCare Laboratory, Pretoria, South Africa in the laboratory.

### Quantitative measures

An Architect 4100 (Immuno) quantitative chemiluminescent microparticle immunoassay was used to test for total testosterone and SHBG, and an Architect 8200 (Chemistry) quantitative Bromocresol purple was used to measure albumin. Total testosterone, albumin, and SHBG were used to calculate the free and bioavailable testosterone concentrations using the calculator available at http://www.issam.ch/freetesto.html following Vermeulen’s formula. Total cholesterol was measured using the Architech 8200 (Chemistry) quantitative enzymatic reaction, whereas the glycerol phosphate oxidative chemical reaction, on the same equipment, was used to measure the triglyceride levels.

### American relevant NHANES data

The National Health and Nutrition Examination Survey (NHANES) is a survey which collected health and nutritional data of the United States population over several years^52^. We sourced a subset of the NHANES testosterone data of men without PCa from Hu et al.^24^, selecting only the age group of 40–59 years (further categorized as younger men) and men ≥ 60 years (further categorized as older men).

### Statistical analyses

The data were analyzed using R software with the following packages: ggpubr and tidyverse (ggplot2) for boxplots and LOESS curves, respectively. The difference between the means of PCa-positive and -negative patients was analyzed using Student’s t test, the Kruskal‒Wallis test and the Wilcoxon test. The Fischer’s exact test was used to assess the difference in testosterone between American men (Black and White) and Black South African men. A *p* < 0.05 value indicated significance. Locally estimated scatterplot smoothing (LOESS) curves were generated to visually examine the relationships between biochemical factors.

## Electronic supplementary material

Below is the link to the electronic supplementary material.

Supplementary Material 1

## Data Availability

The data are available for bona fide researchers upon request to the corresponding authors.
