# Supplementary material for: Associating serum testosterone levels with African ancestral prostate cancer health disparities
Source: Sci Rep. 2025 Apr 8;15:12013. doi: 10.1038/s41598-025-92539-y (PMC11978783; doi:10.1038/s41598-025-92539-y)
Supplement: Supplementary file 1 — Supplementary Material 1 [file 41598_2025_92539_MOESM1_ESM.docx]

**Associating serum testosterone levels with African ancestral prostate cancer health disparities**

Maphuti Tebogo Lebelo, Naledi Mmekwa, Melanie Louw, Weerachai Jaratlerdsiri, Shingai B.A. Mutambirwa, Massimo Loda, Vanessa M. Hayes and M.S. Riana Bornman

**SUPPLEMENTARY DATA**

**
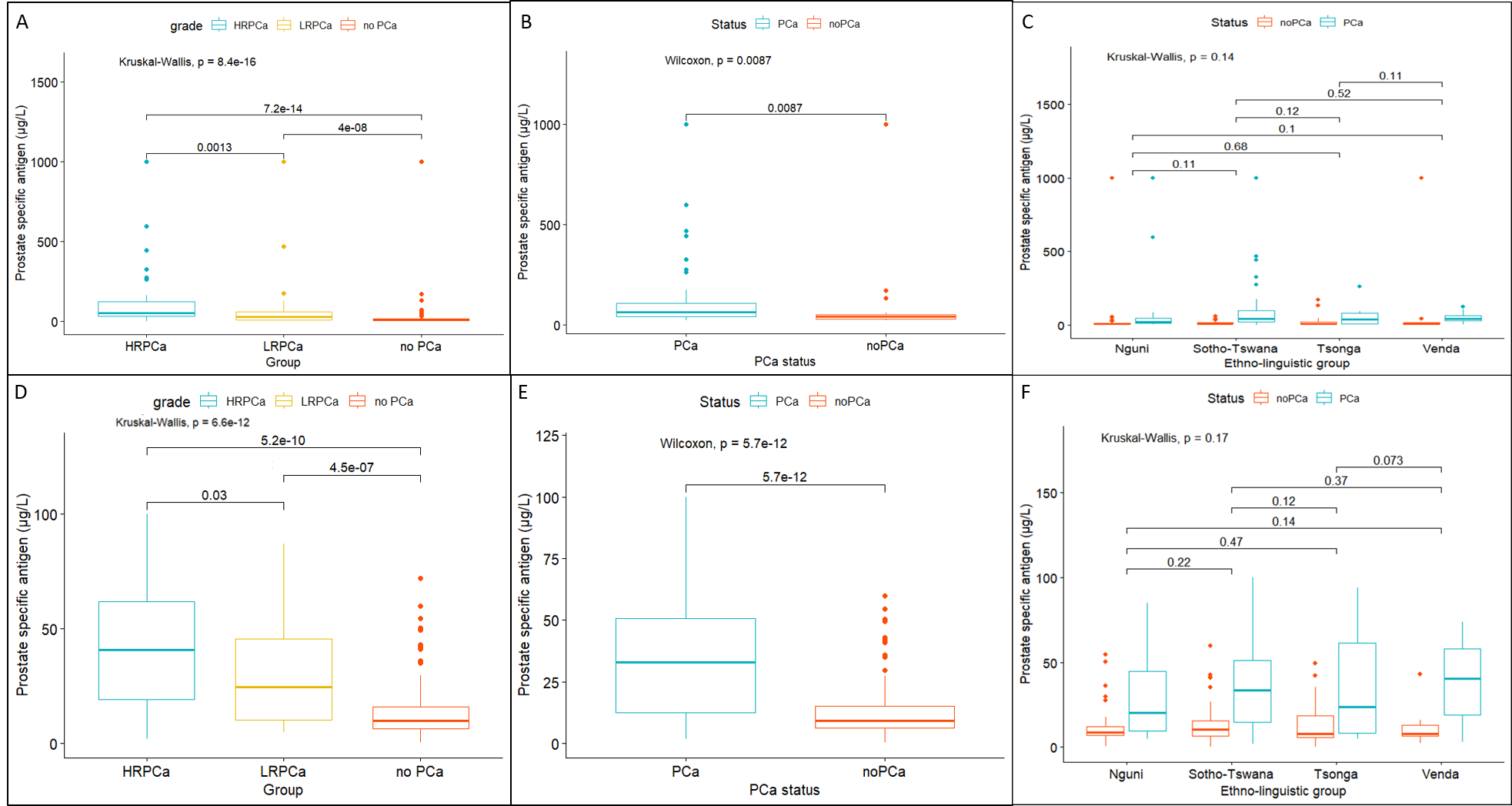
**

**Figure S1**. Boxplots of prostate specific antigen levels of men with and without prostate cancer (PCa). Prostate specific antigen levels (PSA) by **(A)** PCa clinicopathological group including high-risk (HRPCa), low-risk (LRPCa) and no PCa, **(B)** PCa status for those with PSA levels ≥20 µg/L and **(C)** ethnolinguistic group. Excluding for patient outliers defined here as a PSA levels >105 µg/L, PSA levels were redefined by (**D**) PCa clinicopathological group, (**E**) PCa status for those with PSA levels ≥20 µg/L and (**F**) ethnolinguistic group.

**
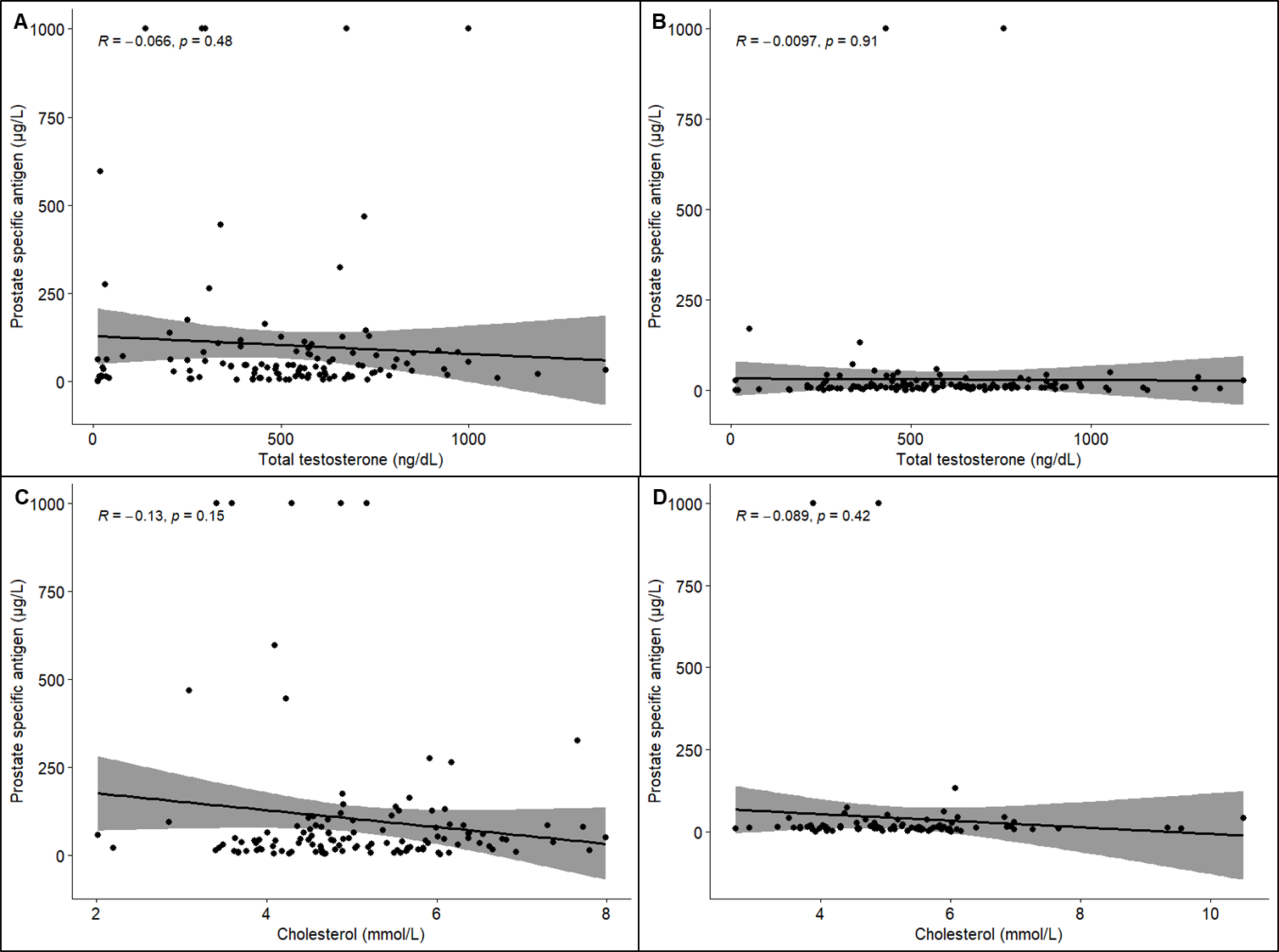
**

**Figure S2:** Scatterplots depicting the correlation between Prostate Specific Antigen (PSA) and testosterone or cholesterol levels for Black South African men by prostate cancer (PCa) status. PSA and testosterone level correlations for **(A)** PCa cases and **(B)** controls, and for PSA and cholesterol level correlations for (**C**) PCa cases and (**D**) controls.

**
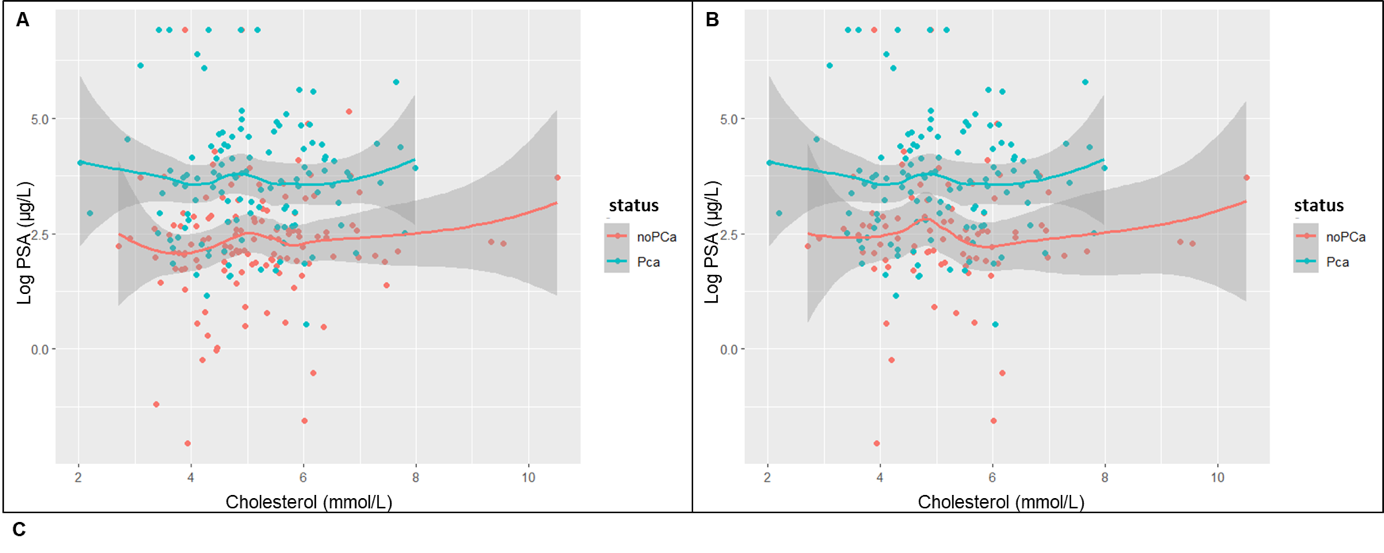
**

**Figure S3:** LOESS curves depicting the relationship between prostate specific antigen (PSA) and cholesterol levels by PCa status. **(A)** LOESS curve of PSA and cholesterol by PCa status. **(B)** LOESS curve of transformed PSA and cholesterol.

**
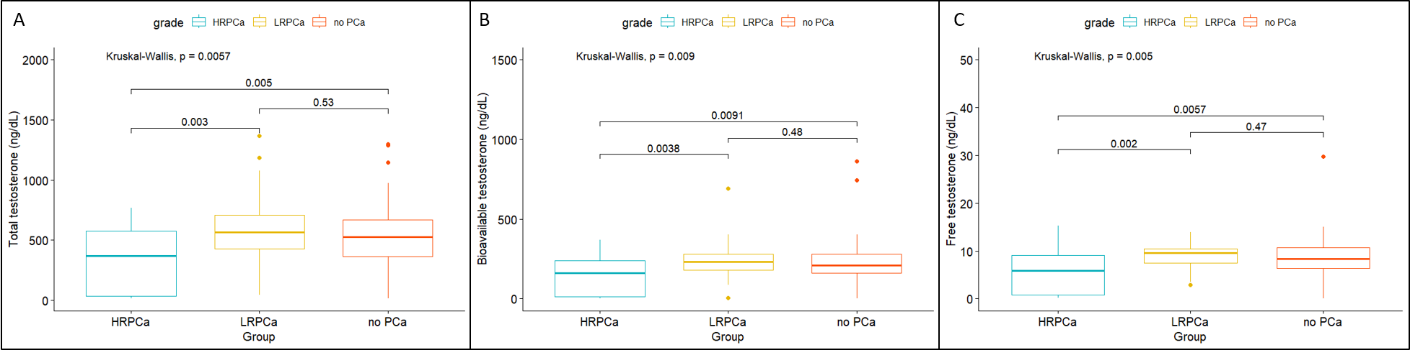
**

**Figure S4:** Boxplots of serum testosterone levels of men ≥65 years, according to prostate cancer (PCa) status in black South African men with (n=82) or without (n=75) prostate cancer (PCa), including high-risk (HRPCa) or low-risk (LRPCa) disease defined by pathology. Boxplot of PCa status and risk for total testosterone **(A)**, bioavailable testosterone **(B)**, and free testosterone **(C)**.

**
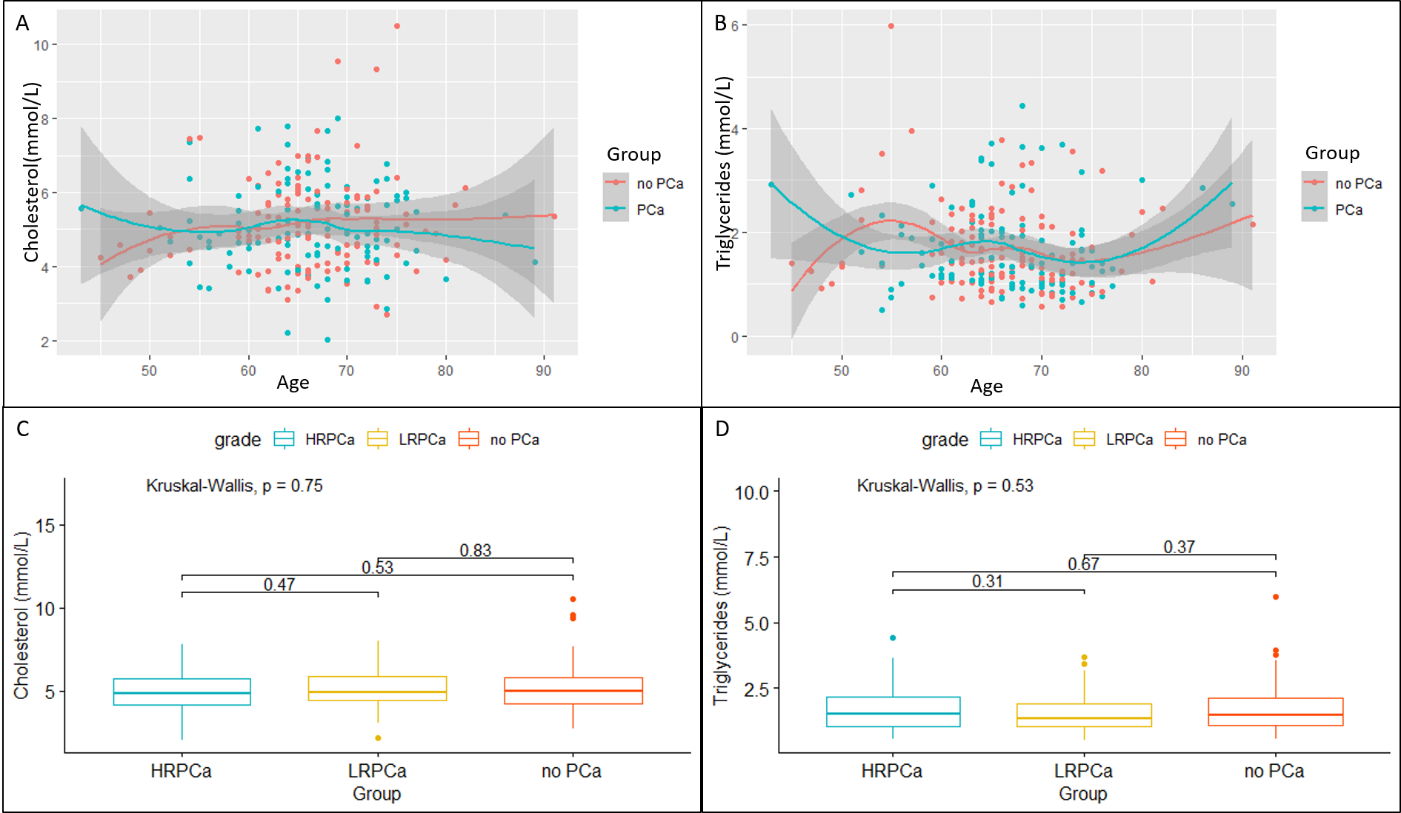
**

**Figure S5:** LOESS curves representing prostate cancer (PCa) status by age for serum cholesterol **(A)** and triglycerides **(B)** levels. Boxplots representing cholesterol **(C)** and triglycerides **(D)** levels by clinicopathological presentation defined as high-risk (HRPCa), low-risk (LRPCa) or no prostate cancer (no PCa).

**
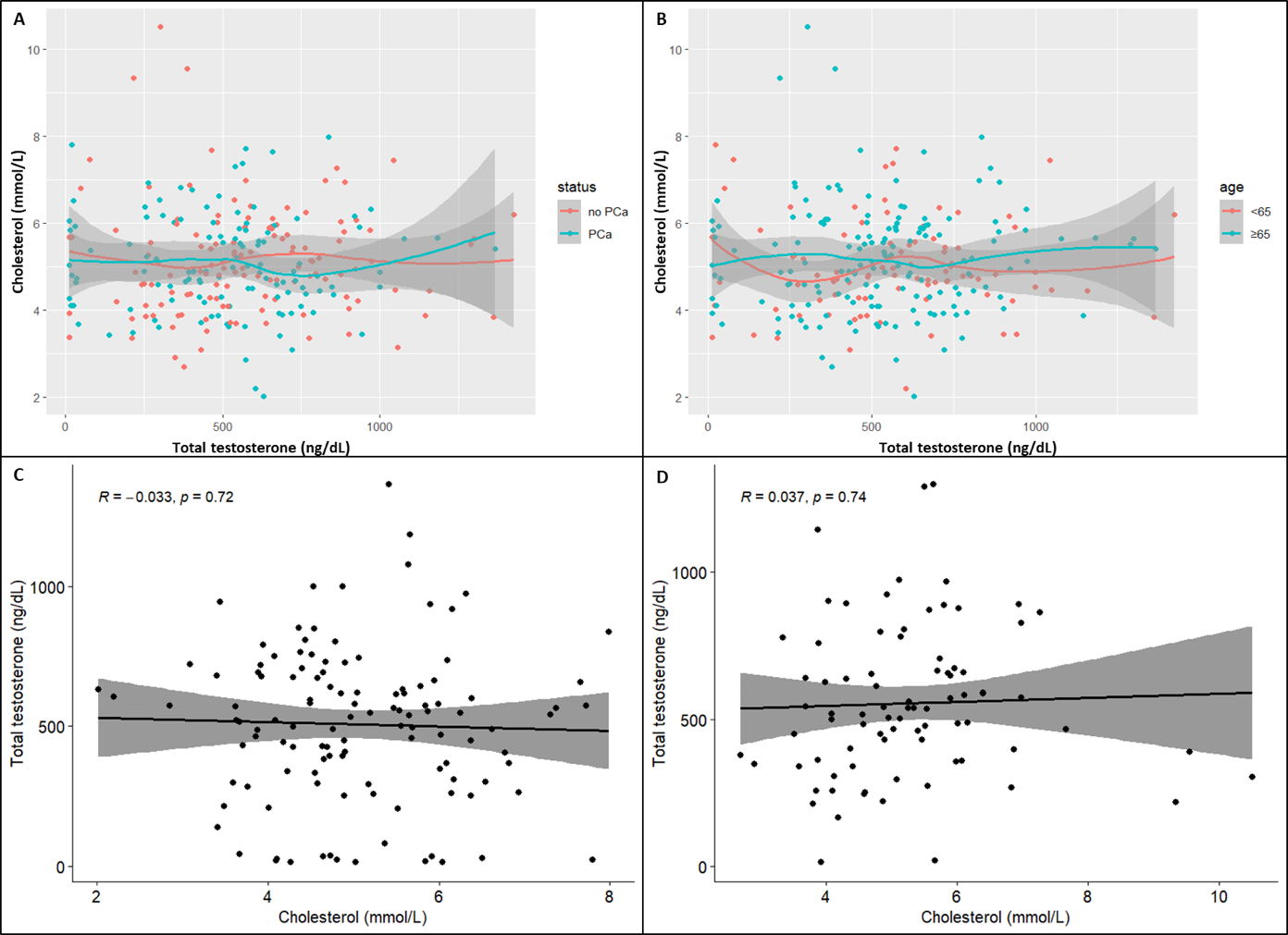

Figure S6:** LOESS curves depicting the relationship between serum cholesterol and total testosterone levels by PCa status **(A)** and age **(B)**. Scatter plots depicting the correlation between serum cholesterol and testosterone in men with PCa **(C)** and without PCa **(D)**.

**Table S1**: Mean testosterone levels in younger (<65 years) and older (≥65 years) Black South African men defined by PCa status.

| **Age** | **40–64 years** | | | **≥65 years** | | |
| --- | --- | --- | --- | --- | --- | --- |
|  | **PCa Controls** (n = 46) | **PCa Cases**  (n = 45) | **Fold^1^** | **PCa Controls** (n = 82) | **PCa Cases**  (n = 75) | **Fold^2^** |
| **Total T** (ng/dL) | 590.45 | 585.70 | 0.99 | 561.89 | 479.55 | 1.17 |
| **Free T** (ng/dL) | 9.41 | 12.54 | 1.33 | 9.50 | 7.37 | 1.29 |
| **Bioavailable T** (ng/dL) | 245.28 | 340.42 | 1.39 | 247.26 | 188.40 | 1.31 |

Abbreviations: PCa: Prostate Cancer; T: Testosterone
Fold^1^: Fold increase between PCa cases and PCa controls
Fold^2^: Fold increase between PCa controls and PCa cases
